# A UK-wide survey of healthcare professionals' awareness, knowledge and skills of the impact of food insecurity on eating disorder treatment

**DOI:** 10.1016/j.eatbeh.2023.101740

**Published:** 2023-04

**Authors:** Carina Kuehne, Amelia Hemmings, Matthew Phillips, Başak İnce, Michelle Chounkaria, Camilla Ferraro, Caroline Pimblett, Helen Sharpe, Ulrike Schmidt

**Affiliations:** a Section of Eating Disorders, Department of Psychological Medicine, King's College London, Institute of Psychiatry, Psychology and Neuroscience, London, UK; b School of Health in Social Science, University of Edinburgh, Edinburgh, UK; c South London and Maudsley NHS Foundation Trust, London, UK

**Keywords:** Food insecurity, Eating disorders, Clinical practice

## Abstract

**Objective:**

Food insecurity (FI) is associated with significant adverse effects on health and well-being and increasingly recognised as a global problem. The current study explored the impact of FI on eating disorder (ED) clinical practice in the UK, aiming to assess healthcare professionals' (HCPs) knowledge, skills and views on the topic of FI in their patients.

**Design:**

This study was an exploratory, mixed-methods, descriptive analysis of online survey data collected from ED HCPs in the UK between September and October 2022.

**Measures:**

A 15-item survey with rating and open-ended questions was circulated to ED professional organisations in the UK. Descriptive statistics were used to summarise quantitative data, including perceived prevalence of FI in ED clinical practice and confidence in knowledge on the topic. Descriptive content analyses provided insight into perspectives on FI screening and aspects to be included in guidance and resources.

**Results:**

93 ED HCPs completed the survey (40.9 % psychologists). Findings demonstrated healthcare providers' limited knowledge on FI and its relation to EDs, while they increasingly perceive it in their patients, as well as a general lack of available resources on how to address FI in ED treatment. HCPs stressed the need for practical guidance and formal training for dealing with FI in their patients, as well as implementing routine screening.

**Conclusion:**

These findings provide both important directions for future research and clinical applications related to screening, assessment, treatment and support of food-insecure patients with EDs.

## Highlights

• UK eating disorder (ED) healthcare professionals (HCPs) increasingly perceive food insecurity (FI) in their patients.
• HCPs know little about the role of FI in ED onset and treatment and stress the lack of available guidance resources.
• HCPs welcome resources on integrating FI into ED care and support introducing routine FI screening in ED services.
• HCPs also expect other cost-of-living crisis driven issues to increasingly impact their patients.

## 1 Introduction

### 1.1 Food insecurity in the UK

The United Kingdom (UK) is experiencing a major cost-of-living crisis. Rising consumer price inflation affects the affordability of nutritious foods, putting healthy diets out of reach for millions (Francis-Devine et al., 2022; The Trussell Trust, 2022a). Consequently, food insecurity (FI), defined as limited access to nutritionally adequate food due to lack of money or other resources (e.g., transport), while not a new problem in the UK, has gained increasing recognition (Environmental Audit Committee, 2019; Loopstra et al., 2019; Yau et al., 2021). FI exists along a continuum of severity ranging from concerns about access to food to extreme hunger.

Rates of FI in the UK have been among the highest in Europe for many years, with nearly 14 % of households having experienced prolonged hunger or had to ration meals due to an inability to afford food in April 2022 (Environmental Audit Committee, 2019). This marks a jump of 57 % in just four months and is mirrored by a record high of food bank usage across the UK (The Food Foundation, 2022; The Trussell Trust, 2022a). FI is exacerbated by rising energy costs, reflected in an increasing demand for non-cook foods at food banks during winter (The Food Foundation, 2022). These data show a concerning trend and suggest FI is a timely topic to investigate.

### 1.2 Evidence on food insecurity and eating disorders

FI has been linked to poor nutritional outcomes and cardiovascular risk factors, including obesity, diabetes and hypertension, which can be largely explained by the relative affordability of energy-dense, low-nutrient foods (Franklin et al., 2012; Gucciardi et al., 2014; Hanson & Connor, 2014; Wells et al., 2020). In addition, the detrimental effects of FI on mental health, including stress, depression, and anxiety have long been established (Arenas et al., 2019; Darling et al., 2017; Maynard et al., 2018). More recently, research has highlighted the impact of FI on the risk of developing disordered eating, characterised by cyclical over- and under-eating depending on food availability (Hazzard et al., 2020; Hazzard et al., 2022a; Hazzard et al., 2022b). This eating pattern, alongside restrictive behaviours such as skipping meals, shows parallels with eating disorder (ED) pathology, making FI an issue of increasing concern in the ED community.

Recent evidence supports cross-sectional associations between FI and self-reported binge eating behaviour, subsequent compensatory behaviours and body dissatisfaction (Altman et al., 2019; Becker et al., 2017; Becker et al., 2019; El Zein et al., 2019; Hooper et al., 2020; Lydecker & Grilo, 2019; Rasmusson et al., 2019; Shankar-Krishnan et al., 2021; Stinson et al., 2018; West et al., 2019). One longitudinal study has demonstrated an association between severe FI and binge eating, suggesting that FI may be a risk factor for EDs (Hazzard et al., 2022a). This is supported by research revealing a relationship between FI and diagnoses of bulimia nervosa and binge eating disorder (Barry et al., 2021; Christensen et al., 2021; Darling et al., 2017; Hooper et al., 2020; Lydecker & Grilo, 2019; Rasmusson et al., 2019; Stinson et al., 2018; West et al., 2019). While further investigation is required, this bingeing behaviour may be driven by food restriction caused by resource constraints, coupled with intermittent availability of cheap, calorific, and highly palatable foods, analogous to animal models of bulimic behaviour (Treasure & Eid, 2019). Alternatively, the relationship between FI and disordered eating may be mediated by the deleterious impact of FI on mood and anxiety symptoms (Dressler & Smith, 2015). Although less consistent, dietary restraint was also found to be associated with FI (Becker et al., 2017; Thompson et al., 2018). Interestingly, weight loss continues to be perceived as a positive outcome even when due to poverty (Taylor et al., 2020), aligning with harmful societal values associating weight with constructs of worth. This links with the seemingly counterintuitive association between FI and compensatory behaviours and suggests moralisation of weight as an underlying explanation.

Taken together, research continues to show an association between FI and ED symptoms, particularly bulimic-spectrum ED pathology. This is concerning given that EDs themselves represent a significant public health problem, with a disease burden comparable to anxiety and depression (Butterfly Foundation, 2012).

### 1.3 Food insecurity in ED clinical practice

ED pathology, by definition, implies difficulties managing eating and weight-related behaviours, making FI particularly relevant to ED treatment. Maintaining regular consumption of nutritious food hinges on patients' consistent food security. As such, ED recovery may become even more challenging for those facing budgetary constraints, given the relative affordability of a poor-quality diet paired with “feast-and-famine” cycles subject to fluctuations in food availability (Hazzard et al., 2020, p. 74). At the same time, food preoccupation as a factor inherent to FI runs contrary to the goal of ED treatment of minimising the dominating role of food in patients' lives (Frayn et al., 2022; Poll et al., 2020).

The majority of FI studies to date have focused on subclinical or non-help-seeking populations, so little is known about the impact of FI on ED treatment. A critical area for future work thus lies in the clinical practice domain. Currently, there is a lack of effective resources or guidance for ED clinicians in the UK – a fact made more concerning by the recent economic recession and increasing ED referrals in the UK post-Covid-19 (Hyam et al., 2023). Additionally, much of the existing research on FI has been conducted in the US and findings may not translate to the UK (Aceves-Martins et al., 2018; Hamnett, 2014; Hood & Norris Keiller, 2016; Loopstra, 2018).

### 1.4 The current study

The current study aimed to explore UK ED healthcare professionals' (HCPs) experiences with FI in their practice and their confidence in providing treatment in this context, as well as to evaluate the awareness of existing guidance resources for FI in ED treatment, or a potential need thereof. Given that other poverty-related issues are likely intertwined with FI, we also aimed to assess HCPs' perspective of their impact to set FI within a wider cost-of-living crisis context.

## 2 Material and methods

### 2.1 Procedure

Data were collected via convenience sampling using a cross-sectional open online survey design between 1 September and 10 October 2022. The survey was advertised in circular emails and on social media channels of relevant professional organisations^3^ in the UK. A link redirected participants to the survey hosted on Qualtrics (https://www.qualtrics.co.uk). Google's reCAPTCHA bot detection was used. Due to the descriptive, exploratory nature of this study, it was aimed to include as many HCPs as possible and no sample size calculation was performed prior to data collection.

Study information and a consent statement were provided on the landing page. Participants were screened for eligibility by confirming they work in healthcare with people with EDs, followed by demographic questions and the main survey. Participation was voluntary, not incentivised, and anonymous. Incomplete surveys were treated as withdrawal of consent, as specified in the consent statement, and thus not included in analyses.

### 2.2 Participants

From 96 completed surveys (64.9 % completion rate), 3 ineligible respondents were removed (due to not working in ED healthcare), leaving a final sample of 93 participants. Detailed demographic characteristics are summarised in Table 1.

**Table 1:** Demographics.

Characteristic	N = 93
Age	
18–24	7 (7.5 %)
25–34	29 (31.2 %)
35–44	27 (29.0 %)
45–54	20 (21.5 %)
55–64	8 (8.6 %)
65–75	2 (2.2 %)
Gender	
Female	82 (88.2 %)
Male	8 (8.6 %)
Not disclosed	3 (3.2 %)
Country	
England	70 (75.3 %)
Scotland	15 (16.1 %)
Wales	8 (8.6 %)
Profession	
Psychologist or psychotherapist	38 (40.9 %)
Nurse or nursing associate	23 (24.7 %)
Dietitian	14 (15.1 %)
Psychiatrist	6 (6.5 %)
Occupational therapist	6 (6.5 %)
Support worker/healthcare assistant	4 (4.3 %)
Speech and language therapist	1 (1.1 %)
Work experience in EDs (years)	
≤2	36 (38.7 %)
3–5	16 (17.2 %)
6–10	14 (15.1 %)
10+	27 (29.0 %)
Healthcare sector	
National Health Service (NHS; public)	87 (93.5 %)
Mixture of NHS and private sector	3 (3.2 %)
Private sector	2 (2.2 %)
Third sector (non-profit, non-governmental)	1 (1.1 %)
Perceived deprivation level of work area	
Moderately to highly deprived	11 (11.8 %)
Mixture of deprived and affluent areas	68 (73.1 %)
Moderately affluent	14 (15.1 %)
Post-code derived Index of Multiple Deprivation (IMD) of work area	
5.00 (most deprived)	7 (7.6 %)
4.00	14 (15.0 %)
3.00	42 (45.2 %)
2.00	11 (11.8 %)
1.00 (least deprived)	16 (17.2 %)
Previous training on FI	
No training, nor done own research	63 (67.7 %)
Done own research	29 (31.2 %)
Formal training	1 (1.1 %)

### 2.3 Measures

#### 2.3.1 Demographics

Sociodemographic variables of interest were profession, Index of Multiple Deprivation (IMD), self-reported perceived deprivation of the work area, years working in the ED field, healthcare sector, and previous training on FI (formal training, own research, neither), (Table 1). The IMD is the official measure of relative deprivation at a small local area level in the UK and is based on a wide range of living conditions (e.g., income, employment, education, crime, housing, services), (Ministry of Housing, 2019). In the current study, the IMD was derived from the service postcode provided by participants and standardised across England, Scotland, and Wales (ranging from 1 [most deprived] to 5 [least deprived]), and was used as proxy measure for the deprivation level of the population attending the service.

#### 2.3.2 Survey

The survey was developed collaboratively by all authors. It included eight rating questions (sliding scales of integers ranging from 0 to 100) and seven open-ended text-based questions asking about participants' encounters with FI in their patients, their knowledge on FI and confidence in discussing it, and the availability of and/or need for resources for managing these issues in ED treatment. An example open question is: “Are there any patient groups within your practice where you are particularly concerned about FI? Please describe.” The full survey is provided as supplementary information (Appendix A).

#### 2.3.3 Analytic plan

Descriptive statistics were calculated to present demographics and key variables related to FI, as well as other poverty-related issues to set FI in a wider context of the UK's cost-of-living crisis. Directed descriptive content analysis was used to analyse optional responses to open-ended, text-based questions (Elo & Kyngäs, 2008; Hsieh & Shannon, 2005). An initial coding framework was developed per question by authors MP and Bİ, capturing common categories, and applied independently to ensure inter-coder reliability. Data were coded case-by-case and disagreements resolved through discussion. Final categories were reviewed and agreed upon by all co-authors.

## 3 Results

### 3.1 Food insecurity in ED practice

**Fig. 1:**
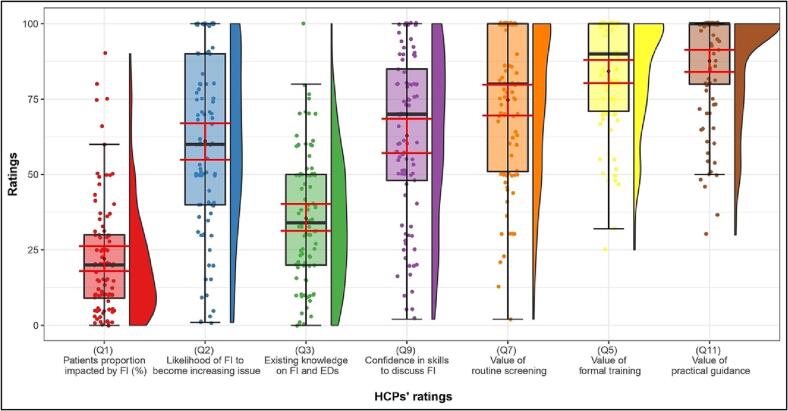
HCPs' ratings of various FI-related issues on 0–100 sliding scales. *Note:* Distributions of HCPs' ratings of FI-related issues on a 0–100 sliding scale ranging from 0 (none/not at all) to 100 (a lot/extremely). Box plots and rain cloud diagrams visualise the jittered raw data, probability density and summary statistics (medians and interquartile ranges; means and 95 % confidence intervals [error bars in red]) of ratings for each survey item. Dependent variables: (Q1) HCP-perceived patient proportion impacted by FI in the past 12 months in %; (Q2) Perceived likelihood of FI becoming an increasing issue over the next 12 months; (Q3) HCPs' reported existing knowledge on FI and EDs; (Q9) HCPs' reported confidence in skills to discuss FI with their patients; (Q7) Belief that FI should be routinely screened for in ED services; (Q5) Perceived helpfulness of formal training; (Q11) Perceived value of practical guidance.

On average, HCPs reported that they perceived more than a fifth of their patients to have experienced FI in the past 12 months (*M* = 22.06 %, *SD* = 19.45) (Fig. 1). HCPs anticipated FI to likely become an increasing issue over the next 12 months (*M* = 61.08, *SD* = 29.07; scale from 0 [extremely unlikely] to 100 [extremely likely]).

**Table 2:** Coded answers for each open-ended, text-based question with frequency of occurrence.

Patient groups HCPs were particularly concerned about	Frequency
	N = 68
Those already struggling financially	38
Certain age groups	26
Those with comorbid diagnoses	15
Those who binge eat	12
Those with restrictive-type eating disorders	12
Patients who are parents	11
Those living alone	9
Families of patients	7
Minoritized groups	5


Advantages and disadvantages of implementing routine screening for FI	Frequency
	N = 83
Advantages	
Assists with a more comprehensive formulation of the person and their ED	43
Greater ability to tailor support and treatment	40
An increased understanding of FI and its link with EDs	14
Reducing stigma through discussing FI in practice	11
Disadvantages	
Worry over the psychological impact on patients	27
Worry over the treatment consequences	20
Lack of knowledge and resources	15


Thoughts on what guidance for HCPs on FI and EDs should include	Frequency
	N = 56
Information about available external support	35
Practical guidance around FI in treatment	31
Sensitive screening and support	18
Education on FI and its link to EDs	15


Additional thoughts about the topic of FI/cost of living	Frequency
	N = 32
The importance of FI and cost-of-living	19
Need to produce guidance around this topic for HCPs	6
The impact of FI and the cost-of-living crisis on HCPs	6

HCPs reporting a higher perceived deprivation of their workplace area seemed to have higher ratings of endorsement of FI being likely to become an increasing issue in their patient population (*n* = 11; *M* = 84.91, *SD* = 23.35) compared to those who perceived their work area as a mixture of deprived and affluent areas (*n* = 68; *M* = 58.62, *SD* = 28.66) or affluent (*n* = 14; *M* = 54.29, *SD* = 27.70). Given the small, unequal group sizes, these findings are preliminary. For a full breakdown of ratings by sociodemographic variables, see Appendix B (Table B1).

Content analysis (Table 2) revealed that when asked to describe the patient groups that HCPs are most concerned about, they most frequently mentioned patients already struggling financially as a group of particular concern. Financial difficulties were commonly mentioned regarding other reported groups of concern, such as parents with dependents, co-occurring diagnoses hindering the ability to work, and age groups with less financial and social resources. Both binge eating- and restrictive-type ED patients were a concern. A more detailed description of categories and illustrative quotes are presented in Appendix C (Table C1).

### 3.2 HCPs' existing knowledge about food insecurity and skills to discuss it with patients

On average, HCPs reported having little existing knowledge about the links between EDs and FI (*M* = 35.51, *SD* = 21.56; scale from 0 [nothing] to 100 [a great deal]), yet reported being moderately confident to discuss FI with patients (*M* = 62.9, *SD* = 27.90; scale from 0 [definitely not] to 100 [definitely yes]), (Fig. 1).

**Fig. 2:**
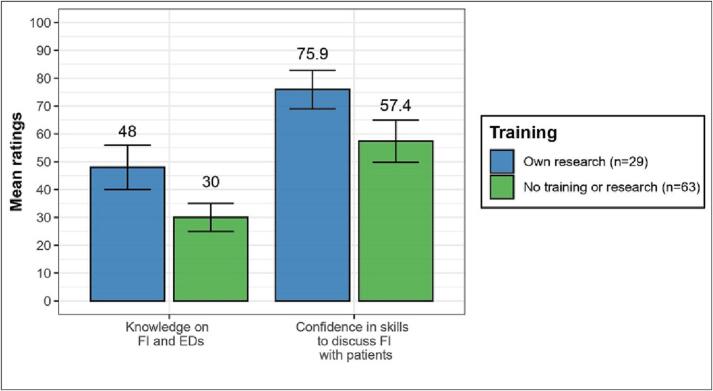
Knowledge and skills by previous training. Note: HCP mean ratings and confidence intervals of knowledge on FI and EDs (ranging from 0 [nothing] to 100 [a great deal]) and confidence to discuss FI with patients (ranging from 0 [definitely not confident] to 100 [absolutely confident]), respectively grouped by having done independent research on the topic vs. having done no research. As only one participant reported having received formal training, this group is not represented in this graph.

Only one participant reported having received formal training as part of their undergraduate degree. Participants who reported having conducted independent research on FI and EDs (*n* = 29) tended to rate their knowledge as higher (*M* = 47.97, *SD* = 20.88) than those who did not (*n* = 63; *M* = 30.00, *SD* = 19.41). The same trend was found for HCPs' confidence in their skills to discuss FI with patients (research: *M* = 75.90, *SD* = 18.19; no research *M* = 56.81, *SD* = 29.80), (Fig. 2). However, given the unequal subsamples, caution is warranted in interpreting these findings.

### 3.3 HCPs' perspectives on routine screening, formal training and practical guidance

HCPs' perspectives on implementing routine screening for FI in ED services were positive (*M* = 74.73, *SD* = 25.27; scale from 0 [definitely not] to 100 [definitely yes]). Content analysis of open-text responses showed that HCPs listed more advantages than disadvantages of FI screening. Advantages included the holistic understanding of patients' psycho-socio-economic position, improved formulation and personalised treatment. In terms of disadvantages, HCPs expressed concerns about consequences for the patients (e.g., shame, embarrassment) and the treatment (e.g., withholding information) due to stigma, though some felt that screening would help to reduce stigma. Some HCPs mentioned a lack of knowledge and resources in mental health services as an obstacle to introducing routine screening (Appendix C, Table C2).

When asked how helpful HCPs would find formal training on FI and its relationship to EDs, ratings were very high (*M* = 84.27, *SD* = 18.0; scale from 0 [not at all helpful] to 100 [extremely helpful]). The value of practical guidance on FI for patients and their carers was rated as equally high (*M* = 87.67, *SD* = 18.69; scale from 0 [definitely not valuable] to 100 [definitely valuable]).

When asked what such guidance should include, HCPs most frequently expressed a need for guidelines around local external resources (e.g., food banks, charities), and practical tips for themselves (e.g., integration of FI into treatment) and their patients (e.g., prioritising nutritious food within stretched budgets). In addition, they highlighted a desire for psychoeducation on the link between FI and EDs as well as information on how to sensitively screen and support patients without inducing shame (Appendix C, Table C3).

### 3.4 Concerns about FI in the broader context of cost-of-living issues

**Fig. 3:**
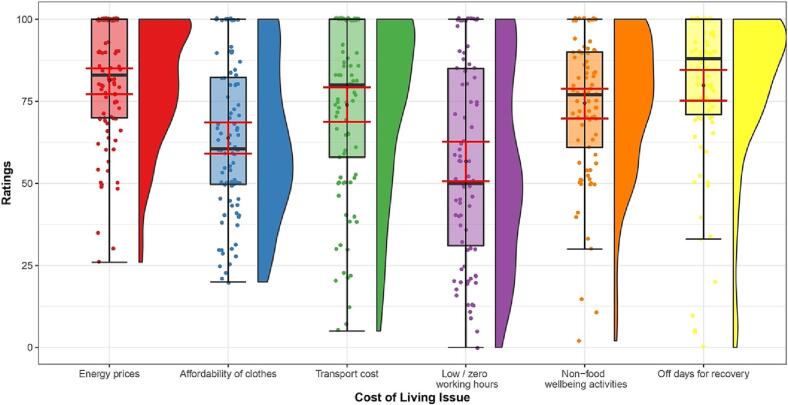
Other cost-of-living issues. *Note:* Distributions of likelihood ratings of other cost-of-living issues to impact ED patients over the next 12 months on a 0–100 sliding scale (0 = definitely not; 100 = definitely yes). Box plots and rain cloud diagrams visualising the jittered raw data, probability density and summary statistics (medians, interquartile ranges; means and 95 % confidence intervals [error bars in red]) of ratings for each cost-of-living issue. Dependent variables: Rising energy costs (e.g., affecting cooking and heating); affordability of clothes (e.g., when needing to change clothes size related to the ED); rising transport costs (e.g., to attend in-person treatment); reliance on zero hours or low-hours contracts; ability to do other, non-food related things that are important for recovery/wellbeing (e.g., hobbies, outings, socialising); ability to take time off work to focus on recovery.

HCPs rated all other presented cost-of-living issues as very likely to impact their patients over the next 12 months (Fig. 3). Rising energy costs were rated the most likely to become an issue for patients (*M* = 81.38, *SD* = 18.30; scale from 0 [definitely not], to 100 [definitely yes]), followed by the inability to take time off work for recovery (*M* = 79.86, *SD* = 23.63) and rising transport costs (*M* = 73.91, *SD* = 26.22).

When asked about further thoughts on FI and the cost-of-living crisis, participants underlined the importance to raise greater awareness and the need to create guidance for HCPs. Some HCPs expressed that they themselves experienced FI, demonstrating the complexity of the issue as it relates to practice (Appendix C, Table C4).

## 4 Discussion

This study builds on the emerging body of research on the issue of FI by assessing its impact on ED clinical practice. Specifically, findings showed that ED HCPs in the UK rate FI as a prevalent and growing concern in their patient population, feel insecure in their knowledge about the links between FI and EDs, and stress the need for practical guidance and resources on the topic.

### 4.1 Food insecurity in ED practice

HCPs reported perceiving over a fifth of their patients to be impacted by FI, surpassing the national average and underlining the concerning relationship between FI and disordered eating (The Food Foundation, 2022). HCPs' anticipated increase in FI maps onto a rising trend on a national level and seems to be even more pronounced in already deprived areas. This is reflected in the widening health inequality across the UK. Those with disabilities, caring responsibilities, receiving universal credit, and from minoritized ethnic groups are disproportionately affected (The Food Foundation, 2022; Yang et al., 2018), yet are the least likely to receive help from public health services (Comber et al., 2011; Healthwatch England, 2022; Huryk et al., 2021). HCPs' equally high rating of other cost-of-living issues likely impacting their patients further confirms that FI appears to exist in the broader context of poverty and may be aggravated by inflated living costs. Particularly for low earners, those without access to social welfare and those already suffering from FI, the ongoing crisis has potentially added to these effects, resulting in a ‘heat or eat’ dilemma currently prevailing in the UK. This is reflected in our qualitative findings, where HCPs noted their concern for patients already struggling financially, including age groups with low social or financial support. The latter finding aligns with research suggesting young people are at disproportionately high risk of FI (McPherson, 2020).

HCPs equally expressed concern for patients with restricting presentations and those with bingeing presentations, highlighting the potential for economic justifications for restriction and concern over food availability hindering recovery. This contrasts most existing research into FI and disordered eating, focusing on bingeing and compensatory behaviours (Hazzard et al., 2022a; Lydecker & Grilo, 2019), and highlights an area deserving of further research.

### 4.2 HCPs' existing skills and knowledge about food insecurity

The contrast between HCPs' limited knowledge about FI and moderate confidence in their skills to discuss it with patients emphasises the need for formal training. Ratings of knowledge about FI and ED and confidence to discuss it tended to be higher in those reporting having conducted independent research on the topic. This might encouragingly suggest the positive impact of FI training and resources for HCPs. However, we cannot be sure of comparability between the resources used and our limited sample size prevents definite conclusions as to significant group differences. In fact, most participants reported no previous education on FI, with only one reporting formal training, yet HCPs seem to increasingly perceive FI in their patients, which further underlines the importance of developing guidance. Accordingly, in open-text responses HCPs stressed a lack of awareness of guidance documents, training, campaigns, or organisations related to the topic of FI for patients with EDs and their carers.

Additionally, HCPs' prevalent perception of the issue in their patients implies active discussions about it in sessions, which represents a surprising finding counterintuitive to the reported limited knowledge of FI. In future explorations, information on whether, how often and to what extent the topic of FI is broached in ED treatment would be informative.

### 4.3 Need for intervention and recommendations

HCPs overall held positive attitudes toward practical interventions for FI. Mirroring findings by Frayn et al. (2022), HCPs felt that more attention needs to be paid to FI in ED treatment. Screening for FI was felt to provide a more complete patient profile, facilitating personalised treatment. Nevertheless, HCPs expressed fear of inducing shame when asking patients to self-disclose FI. Correspondingly, research into food assistance programmes suggested that screening is feasible and acceptable, and that not time and workflow are barriers to implementation, but rather non-disclosure by patients due to shame (Palakshappa et al., 2017). Qualitative research exploring the perspectives of food-insecure individuals has highlighted concerns about actively asking for help (McPherson, 2020). This emphasises the need to train HCPs on how to sensitively broach these issues. Social movements and campaigns to raise awareness of FI in the UK are likely to be beneficial in reducing stigma as a barrier (Sustain, 2021; The Food Foundation, 2020; The Trussell Trust, 2022b; This is Rubbish, 2021).

In general, HCPs welcomed the prospect of formal FI training. Specifically, participants suggested that training should include psychoeducation on the link between FI and EDs, as well as practical tips for sensitive conversation with patients and the integration of FI into ED treatment. Of note, FI is not usually indicative of a lack of individual skills such as ‘poor’ budgeting, but rather structural factors like income. In fact, people display a range of sophisticated strategies to cope with FI, however are often unaware of how to access food support and emergency aid (McPherson, 2020). Consequently, while HCPs should be equipped to assist patients as much as possible, they also need to be made aware of existing food aid provisions and organisations to signpost patients to.

### 4.4 Limitations, implications and future work

Some limitations of the present study must be acknowledged. The self-selected and relatively small sample with unequal subsamples renders conclusions about significant differences in perspectives and experiences across characteristics unwarranted. The fact that there were no respondents from Northern Ireland may be explained by a lack of specialised ED services (Viljoen et al., 2022). Secondly, the standardised IMD may be an imperfect proxy measure of the deprivation of the services' immediate location, not the actual deprivation of the population seen by HCPs. Keeping this in mind, HCPs were asked to indicate the perceived deprivation level of their patient population to achieve a more meaningful measure, although self-report carries its own risk of bias. Thirdly, we did not collect information on the resources used for self-study on FI and EDs. Knowing which resources make HCPs feel more knowledgeable and skilled around FI would aid in developing training and materials and should thus be further explored in future work. Lastly, apart from years of working in the ED field, HCPs were not assessed for their level of training or expertise in ED treatment. It was therefore not possible for us to determine the extent to which the level of training/expertise in the sample was representative of the wider ED HCP workforce in this area.

To our knowledge, this study is the first to explore the impact of FI on ED clinical practice in the UK context. The mixed methods approach allowed a wider scope of an understudied area, resulting in practical recommendations geared toward real change. To develop resources and training, an important next step is to seek the perspectives of food-insecure patients and families to gain a more holistic understanding of the issues and views of what such training should include. Supported by our findings, it is also important to remember that FI exists in the context of poverty more broadly, warranting future investigations of other potentially compounding cost-of-living issues.

## 5 Conclusions

FI is a significant public health concern in the UK. Its detrimental impact on those with EDs is beginning to be recognised, highlighting the need for screening, integration into ED treatments, and sensitive interventions. HCPs' perspectives in this study reflect the state of existing research in this area – not enough attention is paid to FI in ED treatment and guidance, training and resources need to be developed to help HCPs and their patients. Additionally, there is a need for raising wider awareness of this issue to reduce stigma as a barrier to help-seeking. We add our voices to previous calls for investigation into this issue and development of sensible resources and training in this area. However, to target the issue at its centre there is a critical need for a strong policy response to the problem of FI from the UK government. Unless action is taken, FI rates and their adverse effect on EDs are likely to continue to rise.

## Role of funding sources

This work is supported by the Medical Research Council/Arts and Humanities Research Council/Economic and Social Research Council Adolescence, Mental Health and the Developing Mind initiative as part of the EDIFY programme (grant number MR/W002418/1). This paper represents independent research funded by the National Institute of Health Research (NIHR) Maudsley Biomedical Research Centre (BRC) at South London and Maudsley NHS Foundation Trust (SlaM) and King's College London (KCL). The views expressed are those of the authors and not necessarily those of the NIHR or the Department of Health and Social Care.

## Ethical approval

This study was registered at King's College London (reference number: MRA-21/22-33627) and was awarded ethical clearance through the College Research Ethics Committee.

## CRediT authorship contribution statement

**Carina Kuehne:** Conceptualization, Methodology, Investigation, Data curation, Formal analysis (quantitative), Writing – Original Draft. **Amelia Hemmings:** Conceptualization, Methodology, Investigation, Data curation, Formal analysis (quantitative), Writing – Original Draft. **Matthew Phillips:** Methodology, Formal Analysis (qualitative), Writing – Review & Editing. **Başak İnce:** Methodology, Formal Analysis (qualitative), Writing – Review & Editing. **Michelle Chounkaria:** Conceptualization, Methodology, Writing – Review & Editing. **Camilla Ferraro:** Conceptualization, Methodology, Writing - Review & Editing. **Caroline Pimblett:** Conceptualization, Methodology, Writing – Review & Editing. **Helen Sharpe:** Conceptualization, Methodology, Supervision, Writing – Review & Editing. **Ulrike Schmidt:** Conceptualization, Methodology, Supervision, Writing – Review & Editing.

## Declaration of competing interest

The authors have no interests to declare.

## Data Availability

Study data are available on request from the corresponding author. The datasets generated and analysed during this study are not publicly available due to ethical restrictions.

## Appendices Supplementary data

Appendix A Survey of eating disorder clinicians on food insecurity. Appendix A

Appendix B Quantitative tables. Appendix B

Appendix C Qualitative tables. Appendix C

## Footnotes

3 British Eating Disorder Society; ED special interest groups of the Royal College of Psychiatrists and the Royal College of Nursing; British Psychological Society; other independent ED interest groups.
