# Supplementary material for: A UK-wide survey of healthcare professionals' awareness, knowledge and skills of the impact of food insecurity on eating disorder treatment
Source: Eat Behav. 2023 Apr;49:None. doi: 10.1016/j.eatbeh.2023.101740 (PMC10775155; doi:10.1016/j.eatbeh.2023.101740)
Supplement: Appendix A — Survey of eating disorder clinicians on food insecurity. [file mmc1.docx]

# Appendix A: Survey of Eating Disorder Clinicians on Food Insecurity

**The survey that follows will ask you about your knowledge, confidence, and skills in relation to food insecurity in patients with eating disorders.**

Food insecurity means having “**limited access to food** … due to lack of money or other resources”. It is characterised by “limited or uncertain means to access nutritious food in a safe and socially acceptable manner.” Often food insecurity arises from poverty (i.e., not being able to consistently afford food), but it can also arise from not being able to access suitable foods (e.g., because of working or living conditions).

There are different degrees of food insecurity:

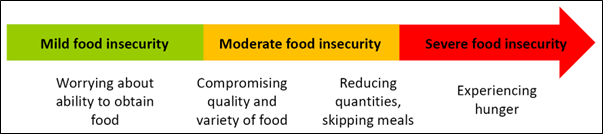


*Source:* Environmental Audit Committee, House of Commons, UK. Available at: <https://tinyurl.com/2p9mpm93>

**In the questions that follow, we would like you to think about the whole spectrum of food insecurity from mild to severe.**

1. Thinking about the past **12 months**, **what proportion (%)** of your patients have been impacted by food insecurity? Please move the slider to indicate your answer.

| 0 | 100 |
| --- | --- |
| ---------------------------------------------------------------\|--------------------------------------------------------------- | |

2. How likely do you think it is that the issue of food insecurity will become **an increasing concern** in your patient population over the next **12 months**?

| Extremely unlikely | Extremely likely |
| --- | --- |
| 0 | 100 |
| ---------------------------------------------------------------\|--------------------------------------------------------------- | |

3. How much do you feel you know about the **links** between food insecurity and eating disorders?

| Nothing at all | A great deal |
| --- | --- |
| 0 | 100 |
| ---------------------------------------------------------------\|--------------------------------------------------------------- | |

4. Have you had any **formal training** on the topic of **food insecurity** and its relationship to mental health and eating disorders?

- Yes, I’ve had formal training.
- I’ve not had formal training, but I’ve done my research
- No, I’ve not had any formal training / done any research myself

Display Question 4.1: If = Yes, I’ve had formal training.

4.1 Please describe your training briefly.

________________________________________________________________

________________________________________________________________

________________________________________________________________

5. How helpful would you find (further) **formal training** on the topic of **food insecurity** and its relationship to mental health and eating disorders?

| Not at all helpful | Extremely helpful |
| --- | --- |
| 0 | 100 |
| ---------------------------------------------------------------\|--------------------------------------------------------------- | |

6. Are there any patient groups where you are **particularly concerned** about food insecurity?

- Yes
- No

Display Question 6.1: If = Yes

6.1 Please briefly describe the **patient groups** you are concerned about and **why**.

________________________________________________________________

________________________________________________________________

7. Do you feel that eating disorder services should **routinely screen** all new patients for food insecurity?

| Definitely not | Definitely yes |
| --- | --- |
| 0 | 100 |
| ---------------------------------------------------------------\|--------------------------------------------------------------- | |

8. In your opinion, what would be the **advantages** and **disadvantages/risks** of a routine screening for food insecurity?

________________________________________________________________

________________________________________________________________

________________________________________________________________

9. Do you feel you have the **confidence or skills** to talk to patients and their families about sensitive issues to do with food insecurity? (e.g., going without food because of lack of money, food bank use, difficulties with shopping, cooking on a restricted budget)

| Definitely not | Definitely yes |
| --- | --- |
| 0 | 100 |
| ---------------------------------------------------------------\|--------------------------------------------------------------- | |

10. Are you aware of any **guidance documents**, **training courses** or **resources**, **campaigns**, or **organisations**  that deal with the topic of **food insecurity** for patients with eating disorders and their carers or more widely for people with mental health problems? Please list them.

________________________________________________________________

________________________________________________________________

________________________________________________________________

________________________________________________________________

11. Do you think some practical guidance on this topic would be of value to **you and your patients**?

| Definitely not | Definitely yes |
| --- | --- |
| 0 | 100 |
| ---------------------------------------------------------------\|--------------------------------------------------------------- | |

12. Please tell us if you have any thoughts of what such guidance should include.

________________________________________________________________

________________________________________________________________

________________________________________________________________

________________________________________________________________

13. Are there any other thoughts you have about the topic of food insecurity that you would like to share with us?

________________________________________________________________

________________________________________________________________

________________________________________________________________

________________________________________________________________

14. Lastly, in addition to food insecurity there are currently a number of **other cost-of-living issues** affecting people in the UK.

How much do you think are these issues likely to impact your patients with eating disorders **over the next 12 months?**

|  | Definitely not | Definitely yes |
| --- | --- | --- |
|  | 0 | 100 |
| a) Rising energy costs (e.g., affecting cooking and heating) | -----------------------\|----------------------- | |
| b) Affordability of clothes (e.g., when needing to change clothes size related to the eating disorder) | -----------------------\|----------------------- | |
| c) Rising transport costs (e.g., to attend in-person treatment sessions) | -----------------------\|----------------------- | |
| d) Reliance on zero hours or low-hours contracts | -----------------------\|----------------------- | |
| e) Ability to do other, non-food related things that are important for recovery / wellbeing (e.g., hobbies, outings, trying new things, socialising) | -----------------------\|----------------------- | |
| f) Taking time off work to focus on recovery | -----------------------\|----------------------- | |

14.1. Please elaborate on any of the aspects from the previous question that you identified as having an **impact on your patients** with eating disorders.

________________________________________________________________

________________________________________________________________

________________________________________________________________

________________________________________________________________

15. Are there any other thoughts you have about the topic of **cost-of-living** that you would like to share with us?

________________________________________________________________

________________________________________________________________

________________________________________________________________

________________________________________________________________
