# Supplementary material for: A UK-wide survey of healthcare professionals' awareness, knowledge and skills of the impact of food insecurity on eating disorder treatment
Source: Eat Behav. 2023 Apr;49:None. doi: 10.1016/j.eatbeh.2023.101740 (PMC10775155; doi:10.1016/j.eatbeh.2023.101740)
Supplement: Appendix B — Quantitative tables. [file mmc2.docx]

# Appendix B: Quantitative tables

## Table B1

Descriptives of food insecurity (FI) ratings by profession, country, healthcare sector, years of experience in eating disorder (ED) field, seniority, previous training, self-reported deprivation level of the work area, and postcode-derived deprivation indices (IMD).

|  |  | **Q1.** | **Q2.** | **Q3.** | **Q9.** | **Q7.** | **Q5.** | **Q11.** |
| --- | --- | --- | --- | --- | --- | --- | --- | --- |
|  |  | **Clinician-perceived patient proportion impacted by FI (%)** | **Likelihood of FI becoming an increasing issue** | **Existing knowledge on FI and EDs** | **Confidence in skills to discuss FI** | **Belief that FI should be routinely screened for in ED services** | **Perceived helpfulness of formal training** | **Perceived value of practical guidance** |
| **Profession** |  |  |  |  |  |  |  |  |
| Psychologists and Psychotherapists | n = 38 | 18.45 (15.09) | 55.03 (31.80) | 34.79 (18.76) | 66.37 (25.96) | 75.03 (22.56) | 88.73 (13.73) | 88.50 (18.19) |
| Nurses and Psychiatrists | n = 29 | 22.72 (24.88) | 64.55 (28.44) | 40.75 (23.61) | 63.76 (30.41) | 76.10 (30.70) | 81.57 (20.57) | 85.83 (21.11) |
| Dietitians | n = 14 | 24.00 (15.25) | 75.14 (25.26) | 31.46 (21.69) | 55.36 (29.79) | 73.29 (26.75) | 81.00 (20.62) | 89.86 (14.93) |
| Allied Health Professionals | n = 11 | 25.55 (15.85) | 54.18 (20.77) | 28.45 (25.39) | 59.00 (27.84) | 75.00 (17.36) | 86.55 (18.02) | 87.36 (14.50) |
| Unspecified | n = 1 | 75.00 (-) | 69.00 (-) | 46.00 (-) | 55.00 (-) | 41.00 (-) | 81.00 (-) | 82.00 (-) |
| **Country** |  |  |  |  |  |  |  |  |
| England | n = 70 | 23.64 (21.15) | 65.76 (27.82) | 38.66 (21.99) | 61.86 (27.06) | 74.26 (25.34) | 85.76 (17.82) | 88.39 (17.12) |
| Scotland | n = 15 | 16.40 (9.16) | 37.87 (23.76) | 29.40 (16.22) | 66.67 (30.48) | 78.13 (24.39) | 76.53 (19.86) | 85.93 (17.72) |
| Wales | n = 8 | 18.88 (16.96) | 63.63 (31.13) | 14.00 (12.26) | 65.00 (33.31) | 72.50 (29.03) | 85.75 (22.81) | 84.63 (27.19) |
| **Healthcare sector** |  |  |  |  |  |  |  |  |
| National Health Service (NHS) | n = 87 | 22.22 (19.67) | 62.28 (28.63) | 35.18 (21.39) | 62.74 (27.74) | 73.86 (25.68) | 84.94 (18.47) | 88.30 (17.86) |
| Mixture of NHS and private | n = 3 | 25.33 (21.50) | 40.67 (35.92) | 42.67 (25.97) | 82.33 (25.58) | 89.67 (17.90) | 81.67 (23.63) | 96.00 (6.93) |
| Private sector | n = 2 | 6.5 (4.95) | 32.50 (38.89) | 31.50 (40.31) | 45.00 (48.03) | 90.00 (14.14) | 56.00 (1.41) | 59.00 (2.83) |
| Third sector | n = 1 | 30.00 (-) | 75.00 (-) | 50.00 (-) | 55.00 (-) | 75.00 (-) | 90.00 (-) | 65.00 (-) |
| **Total** | **n = 93** | **22.06 (19.45)** | **61.08 (29.07)** | **35.51 (21.56)** | **84.27 (18.69)** | **74.73 (25.27)** | **84.27 (18.69)** | **87.67 (18.04)** |

| **ED work experience (years)** | | | | | | | | | | | |
| --- | --- | --- | --- | --- | --- | --- | --- | --- | --- | --- | --- |
| 0-2 | n = 36 | 24.17 (20.33) | 57.25 (27.98) | 33.17 (22.68) | 56.83 (28.53) | 73.14 (26.22) | | 89.47 (15.17) | | 89.78 (16.08) | |
| 3-5 | n = 16 | 14.81 (13.30) | 66 (33.47) | 35.5 (19.97) | 64.25 (26.00) | 77.25 (21.40) | | 83.81 (18.93) | | 87.81 (17.31) | |
| 6-10 | n = 14 | 24.57 (17.66) | 61.57 (32.16) | 36.93 (21.18) | 70.79 (28.94) | 75.50 (22.65) | | 88.50 (15.34) | | 88.93 (20.38) | |
| 10+ | n = 27 | 22.26 (21.95) | 63 (27.10) | 37.88 (22.05) | 66.11 (27.46) | 74.96 (28.38) | | 75.41 (21.76) | | 84.11 (20.07) | |
| **Previous training** | | | | | | | | | | | |
| No training and not done any research | n = 63 | 21.27 (19.59) | 58.59 (27.81) | 30.00 (19.42) | 75.90 (18.19) | 74.08 (25.60) | | 85.46 (19.58) | | 86.73 (19.28) | |
| Done own research | n = 29 | 23.86 (19.71) | 66.52 (31.94) | 47.97 (20.88) | 56.81 (29.80) | 75.28 (24.97) | | 82.17 (16.85) | | 89.28 (15.38) | |
| Formal training | n = 1 | 20.00 | 60.00 | 10.00 | 70.00 | 100.00 | | 70.00 | | 100.00 | |
| **Self-rated perceived deprivation of workplace area** | | | | | | | | | | | |
| Deprived | n = 11 | 32.55 (24.59) | 84.91 (23.35) | 39.80 (27.312) | 49.91 (34.674) | | 85.27 (21.04) | | 87.36 (20.50) | | 89.45 (18.04) |
| Mixture of deprived and affluent areas | n = 68 | 21.75 (18.32) | 58.62 (28.66) | 35.66 (21.456) | 67.28 (25.404) | | 72.07 (24.88) | | 84.41 (17.19) | | 87.37 (18.24) |
| Affluent | n = 14 | 15.36 (18.42) | 54.29 (27.70) | 31.71 (18.273) | 51.86 (29.740) | | 79.36 (28.89) | | 81.14 (24.66) | | 87.71 (18.30) |
| **Deprivation Index – IMD** | | | | | | | | | | | |
| 1.00 (most deprived) | n = 15 | 19.20 (12.08) | 68.93 (26.75) | 28.07 (23.01) | 58.07 (31.173) | | 76.00 (23.59) | | 87.60 (15.519) | | 92.13 (11.48) |
| 2.00 | n = 10 | 23.40 (22.39) | 55.00 (38.71) | 36.20 (20.41) | 62.00 (19.760) | | 83.80 (15.05) | | 76.80 (20.933) | | 72.50 (20.75) |
| 3.00 | n = 40 | 20.43 (18.60) | 59.33 (28.63) | 35.50 (18.93) | 61.85 (29.073) | | 77.33 (26.32) | | 85.18 (16.940) | | 88.98 (17.82) |
| 4.00 | n = 16 | 24.88 (23.94) | 69.81 (24.83) | 45.44 (26.141) | 70.38 (29.523) | | 74.31 (25.51) | | 91.25 (13.767) | | 89.63 (17.90) |
| 5.00 (least deprived) | n = 9 | 20.67 (15.17) | 54.00 (27.79) | 20.25 (12.970) | 59.56 (27.199) | | 54.78 (25.82) | | 72.44 (29.339) | | 87.89 (22.51) |
| **Total** | **n = 90** | **21.37 (18.62)** | **61.78 (28.82)** | **34.82 (21.497)** | **62.52 (28.137)** | | **75.03 (25.22)** | | **84.46 (18.672)** | | **87.68 (18.27)** |
