# Supplementary material for: A UK-wide survey of healthcare professionals' awareness, knowledge and skills of the impact of food insecurity on eating disorder treatment
Source: Eat Behav. 2023 Apr;49:None. doi: 10.1016/j.eatbeh.2023.101740 (PMC10775155; doi:10.1016/j.eatbeh.2023.101740)
Supplement: Appendix C — Qualitative tables. [file mmc3.docx]

**Appendix C: Qualitative tables**

**Table C1.**

Content analysis of open-ended responses to the question ‘Are there any patient groups within your practice where you are particularly concerned about food insecurity?’

| **Category** | **Frequency** | **Description** | **Illustrative Quotes** |
| --- | --- | --- | --- |
| Those already struggling financially | 38 | Food insecurity (FI) was often mentioned to be of the greatest concern for patients and their families who currently struggle financially, including those not in work, those on benefits, those relying on foodbanks, those on low incomes who would struggle to take time off work. This category was often mentioned in tandem with others, for example patients who are single parents on low income and felt to compound the impact. Potential consequences were noted to be not being able to afford food and choosing between heating and eating in the colder months. | *‘Families who may struggle to provide additional food etc for young person with an eating disorder to support recovery.’*  *‘Underweight patients with limited money who cannot afford the food they need to eat to restore weight’*  *‘Patients who are isolated from support networks and solely reliant on state benefits for their income, given the uncertainty over what will happen to benefit rates vis-a-vis rising inflation’* |
| Certain age groups | 26 | FI was felt to impact patients across the life span, but certain age groups were repeatedly mentioned: children and young people (including university students), and the elderly. These groups were often mentioned in the context of financial struggles and inadequate access to social support. | *‘Younger people who have perhaps just moved away from home - university students for example. Not having enough money or know how of what to buy.’*  *‘I worry about sensitive and caring children and young people not wanting to put a burden on their parent and not eating as they should thus increase the incidents of people presenting with an eating disorder.’* |
| Those with  co-occurring diagnoses | 15 | Co-occurring diagnoses were felt to increase risk of FI (or malnourishment) and financial difficulties. Autism was commonly mentioned as a concern, due to associated rigidity of thought and selective eating, as well as those with diagnoses of depression, anxiety, emotional dysregulation. | *‘Patients with Anorexia Nervosa and Autism Spectrum Disorder who can be very fixed on certain brands and therefore face additional challenges in swapping to cheaper alternatives’*  *‘Those with multiple co-morbidities that would make it harder for them to earn a living, require additional support from support network/ government’* |

| **Category** | **Frequency** | **Description** | **Illustrative Quotes** |
| --- | --- | --- | --- |
| Those with binge eating type eating disorders | 12 | FI was said to affect those who binge-eat through increasing guilt and shame around binges, and that financial pressures may lead to binge foods being less-nutritious and lower-cost, as well as increased dietary restriction outside of binges. | *'The cost-of-living crisis may make some feel like they should be ‘cutting back’ on various foods which without support could increase their chances of bingeing and spending large amounts of money on food again impulsively'*  *‘Binge eaters - already spend more than they would like on food, as cost of food increases potential for increased feelings of guilt around 'needing' to binge’* |
| Those with restrictive type eating disorders | 12 | This code relates to those with Anorexia Nervosa (AN) and Avoidant/restrictive food intake disorder (ARFID), where increased restriction was seen to be a consequence of FI. For ARFID, this was proposed to be due to FI further limiting an already limited constrained diet, and for AN, increased restriction was thought to be associated with decreasing access to food and a normalisation of skipping meals. | *‘Hearing others skipping meals/this becoming a social norm may justify some patients with AN also doing this.‘*  *‘ARFID – reduced ability to get nutritionally dense food they will eat’* |
| Patients who are parents | 11 | Clinicians expressed concern that patients with parental responsibilities they may prioritise their children’s and family’s food needs over their own. Single parents with eating disorders were mentioned as of particular concern. | *‘Mothers from deprived areas as they want their kids to eat so will sacrifice possibly their own food for their children’* |
| Those living alone | 9 | Some clinicians proposed that those living alone may be more impacted by FI due to deprived social support networks and limited potential for financial support. | *‘Particularly concerned about those patients who are isolated from support networks’* |
| Families of patients | 7 | Some clinicians were concerned about the families of patients in terms of the financial burden supporting someone through recovery entails. | *‘Families who are supporting a family member with an eating disorder where limited income is impacting on provision of nutritious food for someone who is weight restoring and they have additional costs e.g., transport to appointments’* |
| Minority groups | 5 | A few clinicians noted that those from minority communities, such as BAME individuals, LGBTQ+ individuals, refugees with prior experience of FI, may be disproportionately affected. | *"Black, Asian and Ethnic minority groups more likely to experience food poverty"* |

**Table C2.**

Content analysis of open-ended responses to the question ‘In your opinion, what would be the advantages and risks of a routine screening for food insecurity? ’

| **Category** | **Frequency** | **Description** | **Illustrative Quotes** |
| --- | --- | --- | --- |
| **ADVANTAGES** |  |  |  |
| Assists with a more comprehensive formulation of the person and their eating disorder | 43 | Many clinicians felt that including FI in routine screening would help to gain a more holistic understanding of the person-in-context, in terms of how it may contribute to illness development and/or maintenance, to risk assessment, and to an understanding of a person’s ability to make changes. Screening for FI was also felt to help identify those affected by it who had gone previously unnoticed. | *‘I think it would be helpful in understanding the impact of the systems working around patients that make it difficult for them to challenge their eating disorder. I think it is important to acknowledge additional factors that might make eating challenging.’*  *‘Advantages: full understanding of eating issues - not making assumptions about reasons for restriction. Understanding person’s needs in tandem with social circumstances’* |
| Greater ability to tailor support and treatment | 40 | Closely related to more comprehensive formulations, clinicians felt that screening for FI would allow for more tailored treatment and support for patients, in terms of appropriate meal planning for the patient’s financial situation, identifying potential barriers to recovery, and the ability to signpost to external support. | *‘It would enable us to provide reasonable meal plans that take in to account income, poverty, access to not just food but resources to cook, store food.’*  *‘Helps plan interventions that are appropriate, can provide support to help with food insecurity, makes service more accessible.’* |
| An increased understanding of FI and the link with eating disorders | 14 | Including FI in routine screening was felt by some clinicians to have potential consequences for understanding the prevalence of FI within patient populations, at both a local and national level, and to contribute to increased understanding about the link between FI and eating disorders. | *‘Actively asking about it will give us a better sense of how many of our patients may be at risk/ are experiencing FI.’*  *‘To assess the link between food insecurity and eating disorders would be an advantage.’* |
| Reducing stigma through discussing FI in practice | 11 | Several clinicians noted that making FI a routine part of screening would help to decrease the stigma attached to it. | *‘If it’s a routine screening question it takes the shame and judgement away from asking’*  *‘Mainstreaming these questions can reduce the perception that professionals may be judging or looking down on patients and families’* |

| **Category** | **Frequency** | **Description** | **Illustrative Quotes** |
| --- | --- | --- | --- |
| **DISADVANTAGES** |  |  |  |
| Worry over the psychological impact on patients | 27 | Clinicians commonly worried that discussing FI may induce, shame, embarrassment, guilt, and self-blame, underlining the need to handle discussions sensitively. It was felt that these emotions may become more pronounced when being supported to access food banks for ‘food their eating disorder does not want’. | *‘Disadvantages are families, young people feeling judged/ blamed for unlimited access for food/ guilty for not being able to provide sufficient amounts of food’*  *‘Risk of increased distress, low self-worth with this group, if not handled sensitively.’* |
| Worry over the treatment consequences | 20 | Clinicians expressed concern over the potential negative consequences that screening for FI might have in the treatment, including patients not disclosing the full extent of FI due to actual or feared stigmatisation and ruptures in the therapeutic relationship. | *‘May promote shame and guilt leaving patients and families feeling judged and perhaps unwilling to tell the truth about how much food they can afford during treatment.’*  *‘Could be stigmatising/shaming and damage the therapeutic relationship’* |
| Lack of knowledge and resources | 15 | Some clinicians conveyed a feeling of helplessness over how much they were able to assist with issues arising out of FI in a mental health service, given a lack of service resources, and a lack of knowledge over the external help available. | *‘We need to be in a position to respond (even at least to help formulate) otherwise we risk people feeling like we can help and then nothing gets done’*  *‘Disadvantage as we would need more resources/staff to help support those identified with food insecurity’* |

**Table C3.**

Content analysis of open-ended responses to the question ‘Please tell us if you have any thoughts of what such guidance should include’.

| **Category** | **Frequency** | **Description** | **Illustrative Quotes** |
| --- | --- | --- | --- |
| Information about available external support | 35 | Many clinicians expressed a desire for specific guidance and information about external services in their area that provide support for those with FI, such as charities and food banks. | *‘Contact details of Services that actually help in real time, rather than be put on a waiting list.’*  *‘List of places i.e., food banks etc where people can get support and knowledge around the process for doing this.’* |
| Practical guidance around FI in treatment | 31 | Clinicians commonly wanted guidance to include practical tips for both themselves and their patients. For patients, potential advice surrounded recovery and meal planning on a budget, reducing anxiety caused by FI, and how to prioritise food and eating amidst stretched budgets. For clinicians, desired guidance included how to integrate FI considerations into their practice. | *‘Practical tips suitable for ED patients who may not be motivated to prepare meals/batch cook/want to eat!’*  *‘How to embrace recovery and weight restoration on a budget.’*  *‘When to screen for food insecurity and how often’* |
| Sensitive screening and support | 18 | Noting the stigma and shame that may come with experiencing FI, clinicians wanted guidance to include information about what kinds of questions to ask and how to do this in a sensitive manner. | *‘Thoughts on how to comprehensively screen (what kind of questions). Things that can be particularly helpful/unhelpful with respect to use of language or checklists’*  *‘How to discuss food insecurity with patients who binge and purge without instilling guilt and shame.’* |
| Education on FI, and on the link between FI and eating disorders | 15 | Clinicians expressed that guidance should include educational resources on how FI and eating disorders are linked, for the benefit of both themselves and their patients. | *‘How food insecurity may be manifested in eating disorders; impacts/complications to treatment it presents.’* |

**Table C4.**

Content analysis of open-ended responses to the question ‘Are there any other thoughts you have about the topic of food insecurity/cost of living that you would like to share with us?’

| **Category** | **Frequency** | **Description** | **Illustrative Quotes** |
| --- | --- | --- | --- |
| The importance of FI and cost-of-living | 19 | Clinicians repeatedly mentioned that they felt that FI and the wider cost-of-living crisis were topics of increasing importance that particularly impact those with EDs. They underlined the need to raise awareness of these issues, noting the associated stigma. | *‘I think this topic is already a concern and will only increase in the coming months and years.’*  *‘The link between eating disorders and food insecurity is not in the public domain or to the best of my knowledge being raised at government level’*  *‘The cost of living when recovering from an eating disorder is likely higher than that of an average person without an eating disorder.’* |
| Need to produce guidance around this topic for clinicians | 6 | Some clinicians mentioned the need for clinical guidance on how best to support those with EDs. | *‘Guidance to support clients would be really helpful’*  *‘Seems like a really important topic which many of us professionals need training on.’* |
| The impact of FI and the cost-of-living crisis on clinicians | 6 | Several clinicians also noted the increasing impacts of the cost-of-living crisis on them, including increased personal stress, providing barriers during treatment, and their own FI on their nutrition. | *‘Food insecurity invalidates our ability to care for our patients across multiple domains.‘*  *‘It's not just our patients that it is affecting. Staff are also struggling, and patients will note that we are losing weight....’* |
